# Periodic arrays of plasmonic crossed-bowtie nanostructures interspaced with plasmonic nanocrosses for highly sensitive LSPR based chemical and biological sensing

**DOI:** 10.1039/d0ra09012c

**Published:** 2021-02-18

**Authors:** Abhijit Das, Kamal Kumar, Anuj Dhawan

**Affiliations:** Department of Electrical Engineering, Indian Institute of Technology Delhi Hauz Khas New Delhi 110016 India adhawan@ee.iitd.ac.in

## Abstract

In this paper, we present novel localized surface plasmon resonance (LSPR) sensors based on periodic arrays of gold crossed-bowtie nanostructures interspaced with gold nanocross pillars. Finite difference time domain (FDTD) numerical simulations were carried out to model bulk sensors as well as localized sensors based on the plasmonic nanostructures being proposed. The geometrical parameters of the plasmonic nanostructures are varied to obtain the best possible sensing performance in terms of sensitivity and figure of merit. A very high bulk sensitivity of 1753 nm per unit change in refractive index (nm RIU^−1^), with a figure of merit for bulk sensing (FOM_bulk_) of 3.65 RIU^−1^, is obtained for these plasmonic nanostructures. This value of bulk sensitivity is higher in comparison to previously proposed LSPR sensors based on plasmonic nanopillars and nanocrosses. Moreover, the optimized LSPR sensors being proposed in this paper provide maximum sensitivity of localized refractive index sensing of 70 nm/nm with a FOM_localized_ of 0.33 nm^−1^. This sensitivity of localized refractive index sensing is the highest reported thus far in comparison with previously reported LSPR sensors. It is also demonstrated that the operating resonance wavelengths of these LSPR sensors can be controllably tuned for specific applications by changing the dimensions of the plasmonic nanostructures.

## Abstract 2

Plasmonic nanostructure with very high localized LSPR sensitivity around 1310 nm and 1550 nm communication wavelengths.

## Graphical abstract

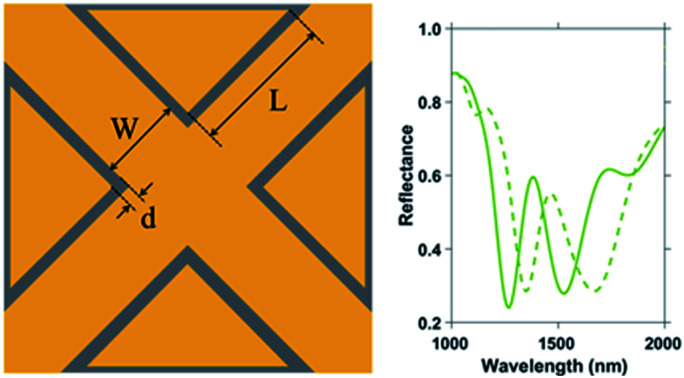

## 1. Introduction

Surface plasmons are collective oscillations of free electrons at metal–dielectric interfaces and can be excited in metallic thin films or gratings as propagating plasmons (surface plasmon polaritons) or in metallic nanoparticles as localized surface plasmons. While several coupling mechanisms such as prism coupling or grating coupling are required to couple the incident light to the surface plasmon polaritons, incident light is directly coupled into localized surface plasmons (LSPs) in metallic nanoparticles. Coupling of the incident radiation to LSPs in metallic nanoparticles or nanostructures leads to enhanced scattering and absorption of the incident light at certain resonant wavelengths and this phenomenon is called localized surface plasmon resonance (LSPR).^1,2^ Sensors based on LSPR have high sensitivity to changes in the localized refractive index in the vicinity of the metal nanoparticles or nanostructures.^3^ Sensors based on LSPR can provide a real-time analysis of label-free biomolecule detection with high sensitivities.^4^ While traditional SPR sensors require bulky setups for coupling of the incident light into surface plasmons (such as the Kretschmann or Otto configurations), the LSPR-based sensors do not require any coupling mechanism as light can be directly coupled into LSPs in nanoparticles and nanostructures.

Over the past few years, different geometries of plasmonic nanostructures have been employed for LSPR-based sensing—nanospheres,^5,6^ triangular nanoplates,^7^ nanoholes,^8–10^ nanorods,^11^ nanorings,^12,13^ nanostars,^14,15^ nanocubes,^16^ nanodiscs,^17^ nanoring resonator array,^18^ among others. Numerous plasmonic systems have reported Fano resonances—nano ring/disk cavity,^19^ bimetallic fanocubes,^20^ split-ring resonator/disk nanocavities,^21^ nanoslits,^22,23^ dual-disk rings,^24^ nanodisks,^25,26^ nanoclusters,^25,27^ nano-dolmen,^28^ nanocylinders,^29^ and nanocubes.^30^ Periodic arrays of nanorings^31–33^ have been investigated for high figure of merit. Multiple layer metal-insulator-metal nanodisks^34^ and bowtie nanoantenna^35^ have been reported. Chiral gold (Au) nanohooks have been presented with circular dichroism measurements for increased refractive index sensitivity.^36^ Alloyed nanodisk arrays of gold–silver have been reported with symmetry breaking-induced mode splitting leading to precisely engineered sensing modes.^37^ Patterned nanotriangle arrays of graded Ag–Cu have also been demonstrated.^38^ A tailored design of a nanoline with a nanocross was used to create a gold plasmonic nanocavity for label-free detection of biomarkers.^39^

In this paper, we propose highly sensitive LSPR sensors based on periodic arrays of gold crossed-bowtie nanostructures interspaced with gold nanocross pillars. These LSPR sensors were modelled using FDTD numerical simulations, and their parameters were optimized to obtain excellent sensing performance both as bulk refractive index sensors and as localized refractive index sensors. The main sensing performance characteristics studied by us were the sensitivity and figure of merit, *i.e.* the ratio of the sensitivity to the full wave half maxima (FWHM) of the plasmon resonance dip in the reflectance spectra from these plasmonic nanostructures. The plasmonic nanostructures being proposed provide a very high value of bulk sensitivity of 1753 nm RIU^−1^ with a figure of merit for bulk sensing (FOM_bulk_) of 3.65 RIU^−1^. In some of the structures proposed by us, values of FOM_bulk_ > 6 RIU^−1^ have also been achieved. This value of bulk sensitivity is higher in comparison to previously proposed LSPR sensors based on plasmonic nanopillars^17^ and nanocrosses.^39^ Moreover, the optimized LSPR sensors being proposed in this paper provide the maximum sensitivity of localized refractive index sensing of 70 nm/nm with a FOM_localized_ of 0.33 nm^−1^. This sensitivity of localized refractive index sensing is the highest reported thus far in comparison with previously reported LSPR sensors. Hence, the plasmonic nanostructures being proposed in this paper can be employed for highly sensitive detection of biomolecules such as nucleic acid biomarkers of infectious diseases, antigens, or antibodies. The plasmon resonance wavelengths of these LSPR sensors can also be tunably varied for a wide spectral range (from 1100 nm to 2500 nm) by changing the geometrical parameters of the plasmonic nanostructures being proposed. This can allow LSPR-based bulk and localized sensing to be carried out at desired wavelengths of interest.

## 2. Structure and simulation details

**Fig. 1:**
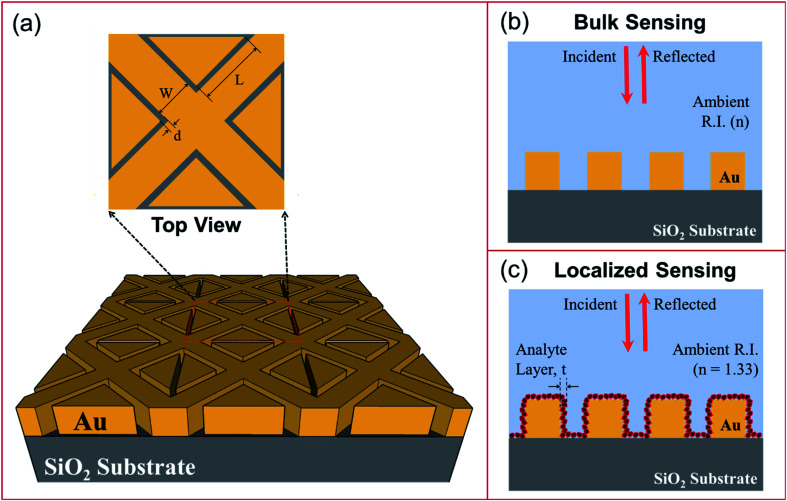
Schematics showing: (a) plasmonic nanostructures consisting of a periodic array of Au crossed-bowtie nanostructures interspaced with Au nanocrosses on a SiO_2_ substrate, with a top view inset; (b) bulk LSPR sensing arrangement using the plasmonic nanostructures; and (c) localized LSPR sensing arrangement. Here, ‘*W*’ is the nanocross pillar width and ‘*d*’ is the edge-to-edge gap distance between all the pillars. The side length of the right-angled triangular pillars is given by ‘*L*’. The height of the nanopillars is 50 nm.

The schematic representation of the LSPR-based sensor being proposed is shown in Fig. 1. The metallic nano-bowties are formed of right-angled isosceles triangular pillars placed pointing towards each other on all sides of a nanocross pillar. This unit structure is repeated along both the *x*- and *y*-axes to simulate a periodic structure. The side length of the isosceles triangles forming the nano-bowties is taken as ‘*L*’, while the width of the nanocross pillar is ‘*W*’. All edge-to-edge gaps ‘*d*’ in the nanostructure geometry have been taken to be the same. The height of all the pillars was fixed at 50 nm. The plasmonic material of the nanocross and nano-bowtie pillars was selected to be gold due to its inert characteristics in interactions with most chemical and biological compounds. The gold pillars were simulated over a silicon dioxide (SiO_2_) substrate. The complex dielectric constants of gold and SiO_2_ were obtained from Palik.

Three-dimensional FDTD simulations of the gold nanopillars on SiO_2_ substrate were carried out using the commercially available FDTD software called Lumerical FDTD Solutions. In the FDTD simulations, plane polarized light was incident on the plasmonic nanostructures by employing a broadband plane wave source—with wavelengths ranging from 300 nm to 3000 nm—placed at a distance of 500 nm above the surface of the substrate. Due to the symmetry of the nanostructure geometry along both *x*- and *y*-axes, the reflection spectra and performance characteristics were found to be the same for both s- and p-polarized light. The top and bottom boundaries of the simulation region were taken to be perfectly matched layers (PML). Convergence studies were carried out to determine the appropriate grid size for which the FDTD simulations are convergent (*i.e.* the simulation results do not change on further decrease of the grid size). A mesh of 1 × 1 × 1 nm^3^ has been taken in the region containing the plasmonic nanostructures for the bulk sensing analysis. For localized sensing analysis, the mesh size was reduced to 0.4 × 0.4 × 0.4 nm^3^ for the region containing the plasmonic nanostructures. For both the bulk sensing analysis and the localized sensing analysis, non-uniform mesh grids were taken in the regions surrounding the plasmonic nanostructures. In the FDTD simulations of the LSPR-based bulk sensors, the ambient refractive index was varied from 1.33 to 1.53 and the reflectance spectra calculated for the different plasmonic nanostructures.^38,40^ In order to model a localized change of refractive index around the plasmonic nanostructures (as would be caused by a biomolecular layer), a 2 nm thin layer of a higher refractive index material (*n* = 1.53) was added over the gold nanopillars, while the material surrounding the 2 nm thin layer was taken to be water (*n* = 1.33).^41,42^ The reflectance spectra of the plasmonic nanostructures were determined by using frequency domain power monitors placed above the plane wave source.

The proposed structure can be fabricated by focused helium ion beam milling (HeFIB).^43,44^ Thin films of plasmonic metals such as Au can be evaporated on SiO_2_ substrates using either electron beam or thermal evaporation. Subsequently using HeFIB, the evaporated gold film can be directly patterned by high energy helium ions. HeFIB using 30 keV helium ions has been employed for the controlled fabrication of sub-10 nm gaps between metallic nanostructures, including gaps as small as 5.5 nm.^44^ In the plasmonic nanostructures being proposed in this paper, very small regions of the gold film need to be milled to form the 6–10 nm gaps between the gold nanopillars. Therefore, even with the low milling rates, HeFIB provides a viable procedure for fabrication of the proposed nanostructure.

Transmission electron microscopes (TEMs) have also been used for nanosculpting metal films^45^*via* Transmission Electron Beam Ablation Lithography (TEBAL). Herein, to fabricate the nanostructures proposed in this paper, gold films evaporated on SiO_2_ substrates can be directly patterned by TEBAL, which has reported sub-10 nm gaps between metallic nanostructures, including gaps as small as 1.5 nm.^45^

## 3. Results and discussion

### 3.1 Bulk LSPR sensing

The performance of the LSPR-based sensor is given by its sensitivity and the figure of merit, FOM_bulk_. The sensitivity of the sensor is evaluated in terms of spectral shift in the plasmon resonance dip wavelength, Δ*λ*, with respect to a change in ambient refractive index, Δ*n*.^46^ In this text, the sensitivity is reported from the slope of the linear fit trendline in the plots for shift in plasmon resonance dip wavelength, Δ*λ*, as a function of the ambient refractive index. The measure of performance of the sensor is also inversely proportional to the FWHM of the plasmon resonance dip because narrower dips are easier to detect. Therefore, the figure of merit for the sensor is given as^39^
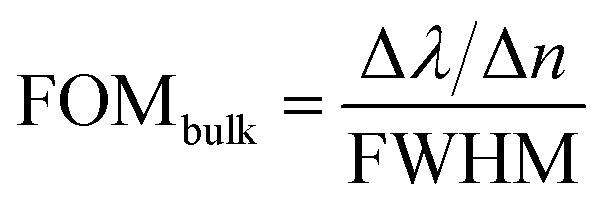


The different parameters of the nanostructure geometry were varied to determine the wavelengths of plasmon resonance dip and the respective performance evaluated. For the analysis of bulk sensing characteristics, the ambient refractive index in the simulation region was varied from *n* = 1.33 to 1.53. The effect of each of the parameters varied was studied individually and is presented accordingly.

#### 3.1.1 Variation in the nanocross pillar width, ‘*W*’

**Fig. 2:**
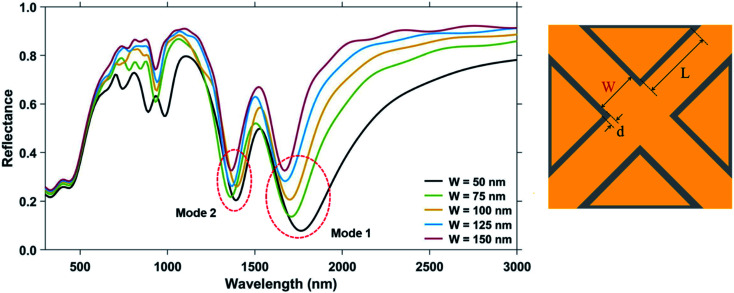
Reflectance spectra of plasmonic nanostructures, consisting of a periodic array of Au crossed-bowtie nanostructures interspaced with Au nanocrosses, for different values of nanocross pillar width, ‘*W*’. The triangular pillar side length, ‘*L*’ and the edge-to-edge gap distance, ‘*d*’ were kept constant at 100 nm and 6 nm, respectively. Ambient refractive index, n was taken to be 1.33. Height of the Au nanopillars was taken to be 50 nm.

For variation in the nanocross pillar width, ‘*W*’ = 50 nm, 75 nm, 100 nm, 125 nm, and 150 nm, the triangular pillar side length, ‘*L*’ and the edge-to-edge gap distance, ‘*d*’ were taken to be constant at 100 nm and 6 nm, respectively. Ambient refractive index was taken as *n* = 1.33. Increasing the nanocross pillar width, increases the size of the nanocross pillars. The reflectance spectra of the nanostructure geometry for varying width of the nanocross pillar, ‘*W*’ is presented in Fig. 2.

Two primary dips are observed for all the nanostructure geometries—which have been named, the Mode 1 and Mode 2 in Fig. 2. For the geometry under consideration, the highest wavelength of plasmon resonance dip is referred to as the Mode 1 plasmon resonance dip. The subsequent dip at a shorter wavelength is the Mode 2 plasmon resonance dip. Higher order plasmon resonance dips were observed at shorter wavelengths. All these plasmon resonance dips – Mode 1, Mode 2 and higher order modes are from hybrid modes generated from the interaction between the crossed-bowtie nanostructures and the nanocross.^39^

The increase in ‘*W*’, leads to a shift in the wavelength of Mode 1 plasmon resonance dip towards the blue end of the visible spectrum (a blue-shift). The change in the width of the nanocross effectively only changes the dimension of the nanocross, without changing the triangles forming the nano-bowties. Now, considering the nanocross to be a combination of two nanorods for the sake of analysis, increasing the width without changing the length essentially reduces the aspect ratio of the constituent nanorods. Reducing the width of a nanorod, while keeping a constant length, leads to a limited transverse movement of the free electrons within the smaller metal volume. As predicted in the theoretical study by Gans, which was later experimentally corroborated,^47^ a decreasing aspect ratio of the nanorods leads to a blue-shift of the plasmon response dips.^48^ The response due to the variation in ‘*W*’ of the proposed nanostructure geometry is dominated by the nature of the response of the nanorods as the geometry of the nanocross changes.

**Fig. 3:**
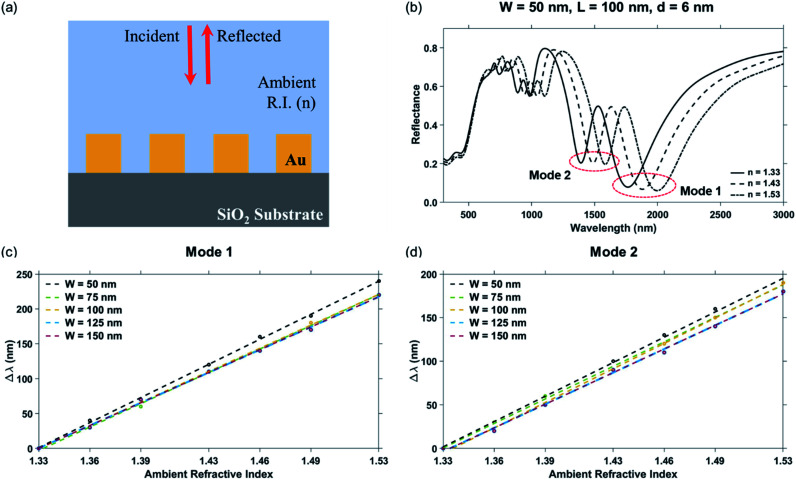
(a) Schematic showing the plasmonic nanostructures consisting of a periodic array of Au crossed-bowtie nanostructures interspaced with Au nanocrosses employed for carrying out bulk refractive index sensing. (b) Shift in the reflectance spectra due to a change in ambient refractive index for the plasmonic nanostructures with nanocross pillar width ‘*W*’ = 50 nm. Shift in dip wavelength as a function of ambient refractive index for: (c) Mode 1 resonance and (d) Mode 2 resonance with different nanocross pillar widths ‘*W*’. The triangular pillar side length, ‘*L*’ and the edge-to-edge gap distance, ‘*d*’ were kept constant at 100 nm and 6 nm, respectively. Height of the Au nanopillars was taken to be 50 nm.

In Fig. 3, the change in the reflectance spectra of the plasmonic nanostructures on varying the ambient refractive index, *n*, is shown for different values of the nanocross pillar width. A linear relationship between the shift in resonance dip and the ambient refractive index is observed for both the Mode 1 and Mode 2 resonances.

The bulk sensing performance characteristics of the nanostructure geometries of different nanocross pillar widths, ‘*W*’ = 50 nm, 75 nm, 100 nm, 125 nm, and 150 nm are tabulated in Table 1. The red-shift in the plasmon resonance dip wavelengths due to an increase in ambient refractive index (*n*) can be observed in both the Mode 1 and Mode 2 resonance dips. As the Mode 1 plasmon resonance dip wavelength increases with a decrease in the nanocross pillar width, an increase in the sensitivity of the nanostructure geometry is observed. This can be attributed to greater shifts in plasmon resonance wavelengths with changes in refractive index occurring at higher wavelengths.^49,50^ A maximum sensitivity of 1194 nm RIU^−1^ is observed for the nanostructure geometry with ‘*W*’ = 50 nm, ‘*L*’ = 100 nm, and ‘*d*’ = 6 nm at Mode 1 dip wavelength of 1760 nm. But as the plasmon resonance dip wavelength increases with a decrease in the nanocross pillar width, the Mode 1 plasmon resonance dip becomes broader and has a much higher FWHM value. Therefore, the figure of merit for the nanostructure geometry decreases with a decrease in ‘*W*’. The Mode 2 plasmon resonance dips occur at shorter wavelengths. Consequently, they have smaller FWHM and therefore a higher FOM_bulk_ as compared to the respective Mode 1 plasmon resonance dip. Maximum FOM_bulk_ of 6.46 RIU^−1^ is observed at the Mode 2 resonance dip wavelength (occurring at *λ* = 1370 nm) of the nanostructure geometry with ‘*W*’ = 150 nm, ‘*L*’ = 100 nm, and ‘*d*’ = 6 nm.

**Table 1:** Bulk sensing performance characteristics of the plasmonic nanostructures—consisting of a periodic array of Au crossed-bowtie nanostructures interspaced with Au nanocrosses—employed to detect changes in the ambient refractive index, for different values of nanocross pillar width ‘*W*’. The triangular pillar side length, ‘*L*’ and the edge-to-edge gap distance, ‘*d*’ were taken to be 100 nm and 6 nm. Height of the Au nanopillars was taken to be 50 nm

Nanocross pillar width, ‘*W*’ (in nm)	50	75	100	125	150
Mode 1 dip wavelength for *n* = 1.33 (nm)	1760	1710	1700	1670	1670
Mode 1 sensitivity (nm RIU^−1^)	1194	1118	1106	1086	1086
Mode 1 dip FWHM for *n* = 1.33	400	290	250	240	180
Mode 1 FOM_bulk_ (RIU^−1^)	2.99	3.86	4.42	4.53	6.03
Mode 2 dip wavelength for *n* = 1.33 (nm)	1390	1360	1400	1370	1370
Mode 2 sensitivity (nm RIU^−1^)	967	935	969	904	904
Mode 2 dip FWHM for *n* = 1.33	160	170	160	150	140
Mode 2 FOM_bulk_ (RIU^−1^)	6.04	5.50	6.06	6.03	6.46

#### 3.1.2 Variation in the triangular pillar side length, ‘*L*’

**Fig. 4:**
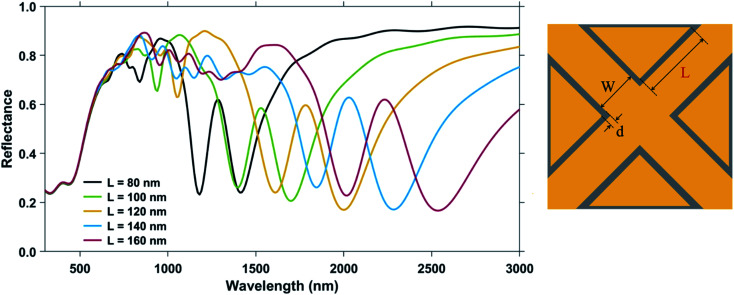
Reflectance spectra of plasmonic nanostructures, consisting of a periodic array of Au crossed-bowtie nanostructures interspaced with Au nanocrosses, for different values of the triangular pillar side length, ‘*L*’. The nanocross pillar width, ‘*W*’ and the gap distance, ‘*d*’ were kept constant at 100 nm and 6 nm, respectively. Ambient refractive index, *n* was taken to be 1.33. Height of the Au nanopillars was taken to be 50 nm.

For studying the effect of variation in the side length ‘*L*’ of the triangular pillar, the values of ‘*L*’ were varied from 80 nm to 160 nm, keeping the nanocross pillar width ‘*W*’, the edge-to-edge gap distance ‘*d*’, and the ambient refractive index *n* to be constant at 100 nm, 6 nm, and 1.33, respectively. By increasing the side length of the isosceles triangular pillars, the area of the nanostructure geometry increases along with the length of the nanocross arms. The reflectance spectra of the different geometries for varying triangular pillar side length is shown in Fig. 4.

The primary Mode 1 and Mode 2 plasmon resonance dips are prominent for all the curves. Higher order modes are more discernible for larger nanostructure geometries. Moreover, as ‘*L*’ increases, the wavelengths of plasmon resonance dip shifts to higher values of the spectrum (red-shift) for all the resonance dips.^39,51^ In the proposed nanostructure geometry, increasing the side length ‘*L*’ of the triangle, increases the length of the nanocross as well. Continuing with the nanorod analysis of the nanocross, we now have here nanorods with a constant width and increasing length, which effectively leads to an increasing aspect ratio of the nanorods. A longer length of the nanorod, keeping the width constant, allows greater longitudinal movement of the free electrons. Increasing the aspect ratio therefore, leads to a red-shift of the plasmon resonance dip.

**Fig. 5:**
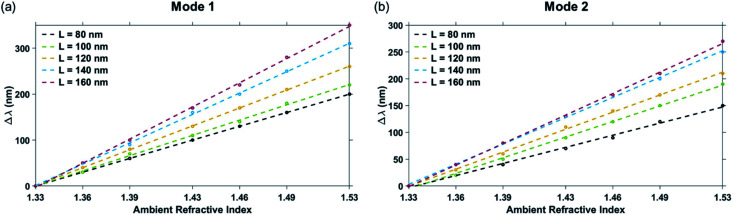
Shift in dip wavelength as a function of ambient refractive index around plasmonic nanostructures consisting of a periodic array of Au crossed-bowtie nanostructures interspaced with Au nanocrosses for: (a) Mode 1 resonance and (b) Mode 2 resonance with different values of triangular pillar side length ‘*L*’. The nanocross pillar width, ‘*W*’ and the edge-to-edge gap distance, ‘*d*’ were 100 nm and 6 nm, respectively. Height of the Au nanopillars was taken to be 50 nm.

The change in the plasmon resonance dip wavelengths (for both Mode 1 and Mode 2) with a change in the ambient refractive index is shown in Fig. 5 for different values of the triangular pillar side length ‘*L*’. The bulk sensing characteristics of the nanostructure geometries with different triangular pillar side lengths, ‘*L*’ = 80 nm, 90 nm, 100 nm, 120 nm, 140 nm, and 160 nm have been tabulated in Table 2. The plasmonic nanostructure having geometrical parameters ‘*W*’ = 100 nm, ‘*L*’ = 90 nm, and ‘*d*’ = 6 nm is particularly significant due to its operability near the 1310 nm and 1550 nm communication wavelength regimes, *i.e.* these nanostructures have plasmon resonance dips around these wavelengths. Both Mode 1 and Mode 2 plasmon resonance dips shift to greater wavelength values as the side length, ‘*L*’ increases. The sensitivity of the nanostructure geometry can be correlated with the plasmon resonance dip wavelength—as the value of the dip wavelength increases with ‘*L*’, so does the sensitivity. This can be explained on the basis of greater shifts in plasmon resonance wavelength (on changing the refractive index of the surrounding medium) occurring at higher wavelengths.^49^ Maximum sensitivity of 1753 nm RIU^−1^ is obtained for the nanostructure geometry with ‘*W*’ = 100 nm, ‘*L*’ = 160 nm, and ‘*d*’ = 6 nm for the Mode 1 plasmon resonance dip wavelength (occurring at *λ* = 2540 nm) with FOM_bulk_ of 3.65 RIU^−1^. On the other hand, the figure of merit mostly decreases even though the sensitivity increases with ‘*L*’, due to a greater increase in the FWHM (from the broadening of the dips) as ‘L' is increased. Highest FOM_bulk_ of 6.85 RIU^−1^ was observed in the Mode 2 plasmon resonance dip of the nanostructure geometry with ‘*W*’ = 100 nm, ‘*L*’ = 80 nm, and ‘*d*’ = 6 nm.

**Table 2:** Bulk sensing performance characteristics of the plasmonic nanostructures—consisting of a periodic array of Au crossed-bowtie nanostructures interspaced with Au nanocrosses—employed to detect changes in the ambient refractive index, for different values of triangular pillar side length ‘*L*’. The nanocross pillar width, ‘*W*’ and the edge-to-edge gap distance, ‘*d*’ were taken to be 100 nm and 6 nm. Height of the Au nanopillars was taken to be 50 nm

Triangular pillar side length, ‘*L*’ (in nm)	80	90	100	120	140	160
Mode 1 dip wavelength for *n* = 1.33 (nm)	1410	1530	1700	2000	2280	2540
Mode 1 sensitivity (nm RIU^−1^)	1000	1000	1106	1301	1549	**1753**
Mode 1 dip FWHM for *n* = 1.33	180	210	250	320	360	480
Mode 1 FOM_bulk_ (RIU^−1^)	5.56	4.76	4.42	4.07	4.30	3.65
Mode 2 dip wavelength for *n* = 1.33 (nm)	1180	1270	1400	1610	1840	2020
Mode 2 sensitivity (nm RIU^−1^)	753	839	969	1065	1247	1339
Mode 2 dip FWHM for *n* = 1.33	110	130	160	210	200	230
Mode 2 FOM_bulk_ (RIU^−1^)	6.85	6.45	6.06	5.07	6.24	5.82

#### 3.1.3 Variation in the gap distance, ‘*d*’

**Fig. 6:**
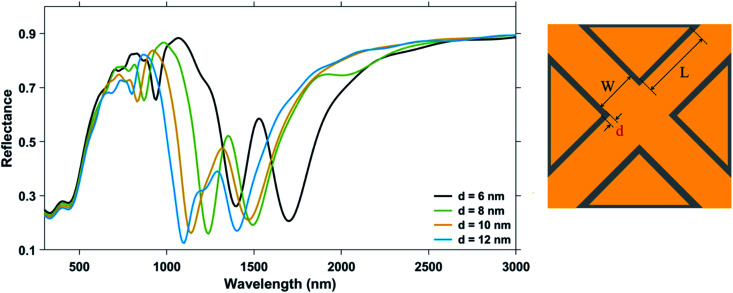
Reflectance spectra of plasmonic nanostructures, consisting of a periodic array of Au crossed-bowtie nanostructures interspaced with Au nanocrosses, for a change in edge-to-edge gap distance, ‘*d*’. The nanocross pillar width, ‘*W*’ and the triangular pillar side length, ‘*L*’ were kept constant at 100 nm and 100 nm, respectively. Ambient refractive index, *n*, was taken to be 1.33. Height of the Au nanopillars was taken to be 50 nm.

The edge-to-edge gap distance was varied as ‘*d*’ = 6 nm, 8 nm, 10 nm, and 12 nm, keeping both the nanocross pillar width, ‘*W*’ and the triangular pillar side length, ‘*L*’ constant at 100 nm. Ambient refractive index, *n* was taken to be 1.33. The combined area of the nanopillars does not change as both the parameters used to set their size—‘*W*’ and ‘*L*’, are not changing. As the separation ‘*d*’ between the nanopillars increases, it effectively increases the area of the simulated nanostructure geometry. The reflectance spectra of the nanostructure geometries with different gap distances are shown in Fig. 6.

**Fig. 7:**
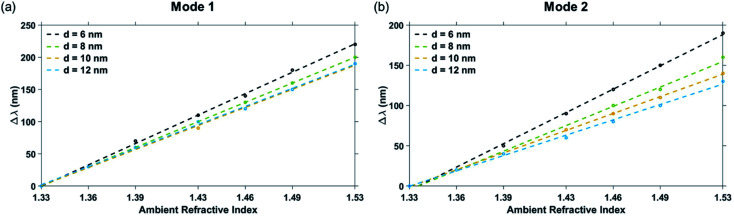
Shift in dip wavelength as a function of ambient refractive index around plasmonic nanostructures consisting of a periodic array of Au crossed-bowtie nanostructures interspaced with Au nanocrosses for: (a) Mode 1 resonance and (b) Mode 2 resonance with different values of edge-to-edge gap distance ‘*d*’. The nanocross pillar width, ‘*W*’ and the triangular pillar side length, ‘*L*’ were 100 nm and 100 nm, respectively. Height of the Au nanopillars was taken to be 50 nm.

The Mode 1, Mode 2 and additional higher modes of resonance are observed in all the curves of Fig. 6. A blue-shift was observed in the wavelengths of plasmon resonance dip, with an increase in the edge-to-edge gap distance between the pillars.^27^ The variation of the ambient refractive index of the nanostructure geometry leads to a shift in their plasmon resonance dip wavelength, which is presented in Fig. 7. The bulk sensing characteristics obtained from the reflectance spectra of the plasmonic nanostructures shown in Fig. 7 for different values of the edge-to-edge gap distance ‘*d*’ have been tabulated in Table 3. The wavelength of plasmon resonance dip red-shifts as the ambient refractive index is increased from *n* = 1.33 to 1.53. It can be observed from Fig. 7 that the nanostructure geometry with the smallest gap ‘d' has the highest sensitivity. This can be attributed to higher EM fields in the gap region for smaller values of gap ‘*d*’. Moreover, as the gap between the nanostructures is decreased, there is a decrease in the restoring force acting on the conduction band electrons of the plasmonic metal. This leads to a decrease in the plasma frequency, which further leads to an increase in the plasmon resonance wavelength associated with the nanostructures. As the plasmon resonance dips occurring at higher wavelengths show greater bulk refractive index sensitivity,^49^ a decrease in the gap distance ‘*d*’ leads to an increase in the sensitivity. We can observe from Table 3 that there is a significant drop in the sensitivity of the nanostructure geometries with an increase in the gap distance ‘*d*’, while there is only a moderate variation in the value of FWHM. Hence, the highest Mode 1 sensitivity of 1106 nm RIU^−1^ with a figure of merit of 4.42 RIU^−1^ is obtained for the nanostructure geometry having the smallest gap distance (*i.e.* for ‘*d*’ = 6 nm). The highest Mode 2 sensitivity of 969 nm RIU^−1^ with a figure of merit of 6.06 RIU^−1^ is also obtained for ‘*d*’ being 6 nm.

**Table 3:** Bulk sensing performance characteristics of the plasmonic nanostructures—consisting of a periodic array of Au crossed-bowtie nanostructures interspaced with Au nanocrosses—employed to detect changes in the ambient refractive index, for different values of edge-to-edge gap distance ‘*d*’. The nanocross pillar width, ‘*W*’ and triangular pillar side length, ‘*L*’ were taken to be 100 nm and 100 nm, respectively. Height of the Au nanopillars was taken to be 50 nm

Gap distance, ‘*d*’ (in nm)	6	8	10	12
Mode 1 dip wavelength for *n* = 1.33 (nm)	1700	1490	1470	1400
Mode 1 sensitivity (nm RIU^−1^)	1106	1000	935	935
Mode 1 dip FWHM for *n* = 1.33	250	220	240	210
Mode 1 FOM_bulk_ (RIU^−1^)	4.42	4.55	3.90	4.45
Mode 2 dip wavelength for *n* = 1.33 (nm)	1400	1240	1140	1100
Mode 2 sensitivity (nm RIU^−1^)	969	767	699	634
Mode 2 dip FWHM for *n* = 1.33	160	130	170	220
Mode 2 FOM_bulk_ (RIU^−1^)	6.06	5.90	4.11	2.88

#### 3.1.4 Plasmon resonance modes

**Fig. 8:**
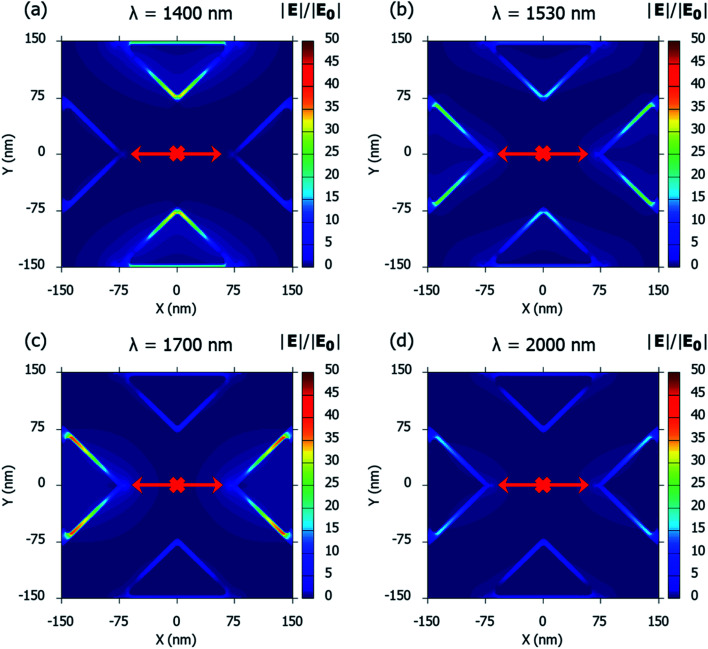
Spatial distribution of the electric-field enhancements around the plasmonic nanostructures, consisting of a periodic array of Au crossed-bowtie nanostructures interspaced with Au nanocrosses, with ‘*W*’ = 100 nm, ‘*L*’ = 100 nm, ‘*d*’ = 6 nm, and the ambient refractive index, *n* = 1.33 for: (a) Mode 2 plasmon resonance dip at *λ* = 1400 nm, (b) off-resonance dip at *λ* = 1530 nm, (c) Mode 1 resonance dip at *λ* = 1700 nm, and (d) off-resonance tail at *λ* = 2000 nm.

The plasmon resonance dips have been further established through spatial distributions of the electric-field enhancements around the plasmonic nanostructures. Fig. 8(a) shows the spatial distribution of the electric-field enhancement around the plasmonic nanostructures described in this paper at the Mode 2 plasmon resonance dip (*λ* = 1400 nm) while Fig. 8(c) shows the distribution at the Mode 1 plasmon resonance dip (*λ* = 1700 nm). The spatial distributions of the electric-field enhancement at the peak frequency (*λ* = 1530 nm) between the resonance modes is shown in Fig. 8(b). Finally, for wavelengths beyond the Mode 1 resonance, an off-resonance field profile at *λ* = 2000 nm is shown in Fig. 8(d).

### 3.2 Localized LSPR sensing

In localized LSPR sensing, it is assumed that the plasmonic nanostructures (periodic array of Au crossed-bowtie nanostructures interspaced with Au nanocrosses) are coated with a 2 nm thick layer of a biomolecule (analyte) having a higher refractive index as compared to the solvent (ambient refractive index). The sensitivity of a localized LSPR sensor is therefore evaluated as the spectral shift in plasmon resonance dip wavelength, Δ*λ* with respect to the thickness of the analyte layer, ‘*t*’ for a given non-changing background refractive index, *n*.^52^ The equivalent figure of merit of a localized LSPR sensor is given by^53^
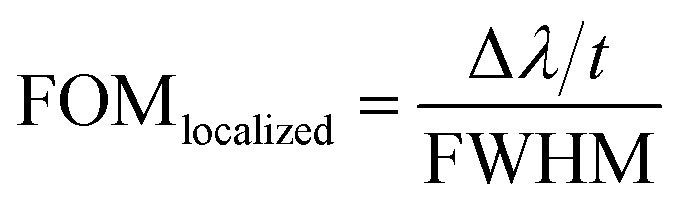


**Fig. 9:**
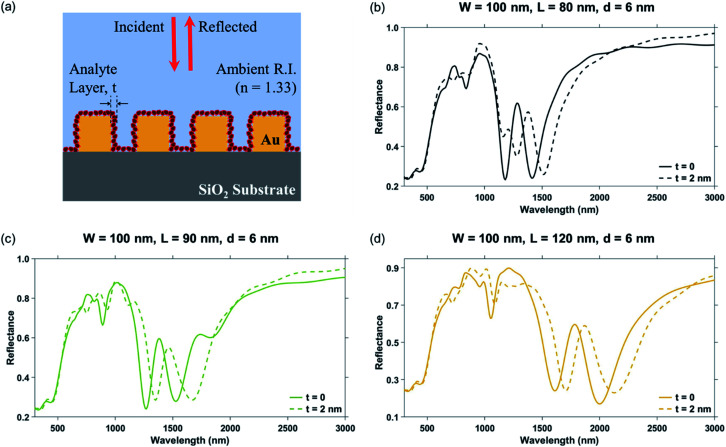
(a) Schematic showing the plasmonic nanostructures consisting of a periodic array of Au crossed-bowtie nanostructures interspaced with Au nanocrosses employed for localized sensing. Shift in the reflectance spectra due to the addition of a 2 nm biomolecule layer having a refractive index of 1.53 over the plasmonic nanostructures, for different values of the triangular pillar side length ‘*L*’: (b) ‘*L*’ = 80 nm, (c) ‘*L*’ = 90 nm, and (d) ‘*L*’ = 120 nm. The nanocross pillar width, ‘*W*’ and the edge-to-edge gap distance, ‘*d*’ were taken to be 100 nm and 6 nm, respectively. Ambient refractive index, *n* was taken to be 1.33. Height of the Au nanopillars was taken to be 50 nm.

The plasmonic nanostructures with nanocross pillar width, ‘*W*’ = 100 nm, triangular pillar side length, ‘*L*’ = 80 nm, 90 nm, and 120 nm, edge-to-edge gap distance, ‘*d*’ = 6 nm, and ambient refractive index, *n* = 1.33 were considered for their wavelengths of plasmon resonance dip and performance characteristics such as sensitivity of localized LSPR sensing (Δ*λ*/*t*) and FOM_localized_. Sensitivity was evaluated for an additional 2 nm layer of a material with higher refractive index, *n*_*L*_ = 1.53 over the Au nanopillars. The reflectance spectra of the nanostructure geometries in the absence and presence of the 2 nm higher refractive index material layer is shown in Fig. 9. As discussed previously in Section 3.1.2, both the Mode 1 and the Mode 2 plasmon resonance dips have a red-shift, as the side length ‘*L*’ increases, the data for which has been tabulated in Table 4. Highest sensitivity of 70 nm/nm is obtained for the nanostructure geometry with ‘*W*’ = 100 nm, ‘*L*’ = 90 nm, and ‘*d*’ = 6 nm at the Mode 1 plasmon resonance dip wavelength (occurring at a wavelength of 1530 nm).

**Table 4:** Localized sensing performance characteristics of the plasmonic nanostructures—consisting of a periodic array of Au crossed-bowtie nanostructures interspaced with Au nanocrosses—employed to detect localized changes in the refractive index caused by the addition of a 2 nm biomolecule layer of *n* = 1.53 over the nanopillars, for different values of triangular pillar side length ‘*L*’. The nanocross pillar width, ‘*W*’ and the edge-to-edge gap distance, ‘*d*’ were taken to be 100 nm and 6 nm. Ambient refractive index, *n* was taken to be 1.33. Height of the Au nanopillars was taken to be 50 nm

Triangular pillar side length, ‘*L*’ (in nm)	80	90	120
Mode 1 dip wavelength for *n* = 1.33 (nm)	1410	1530	2000
Mode 1 sensitivity (nm nm^−1^) for *t* = 2 nm, and Δ*n* = 0.2	50	70	65
Mode 1 dip FWHM for *n* = 1.33	150	210	260
Mode 1 FOM_localized_ (nm^−1^)	0.33	0.33	0.25
Mode 2 dip wavelength for *n* = 1.33 (nm)	1180	1270	1610
Mode 2 sensitivity (nm nm^−1^) for *t* = 2 nm, and Δ*n* = 0.2	50	40	50
Mode 2 dip FWHM for *n* = 1.33	90	130	170
Mode 2 FOM_localized_ (nm^−1^)	0.56	0.31	0.29

Table 5 shows a performance comparison of the proposed nanostructure with that of published plasmonic nanoparticle and nanostructure literature. It can clearly be observed from Table 5 that the plasmonic nanostructures being proposed provide a very high value of bulk sensitivity of 1753 nm RIU^−1^ (with a fairly high figure of merit for bulk sensing (FOM_bulk_) of 3.65 RIU^−1^), which is higher in comparison to previously proposed nanopillar based.

**Table 5:** Characteristics of published plasmonic sensors

Nanostructure^(ref)^	Sensitivity (bulk) (nm RIU^−1^)	FOM_bulk_ (RIU^−1^)	Sensitivity (localized) (nm nm^−1^)	FOM_localized_ (nm^−1^)
Heptamer nanohole array^59^	400	—	2	
Heptamer nanospheres^55^	514	10.6		
Nanobipyramid^60^	540	4.5	—	—
Metamaterial^61^	588	3.8	—	—
Nanostar^62^	879	10.7	—	—
Nanoellipsoid^63^	∼450	∼9.5	∼8	∼0.15
Nanocross^39^	1140	5	—	—
Capped nanoslits^23^	—	—	2.58	—
Nanorings^54^	1300	5	12	0.035
Functionalized nanorods^58^	—	—	12.34	—
Nanocube patch antenna^57^	—	—	22	—
Proposed nanostructure	1753	3.65^a^–6.85	70	0.33

a FOM_bulk_ = 3.65 RIU^−1^ when bulk sensitivity is 1753 nm RIU^−1^.

LSPR sensors such as those based on plasmonic nanorings^54^ and Fano resonances.^39,55^ Although some previously reported LSPR sensors, including those based on Fano resonances,^39,55^ have provided values of FOM_bulk_ as high as ∼10.6 RIU^−1^, the values of bulk sensitivity of those sensors are much lower than that reported in our paper. We also observe from Table 5 that the optimized LSPR sensors being proposed in this paper provide the maximum sensitivity of localized refractive index sensing of 70 nm/nm with a FOM_localized_ of 0.33 nm^−1^. This value sensitivity of localized refractive index sensing is the highest reported thus far in comparison with previously reported LSPR sensors,^56^ at least 3 times higher in comparison with nanopillar based plasmonic sensors^57,58^ and ∼27 times higher than those reported for plasmonic sensors based on Fano resonances.^23,59^ Moreover, LSPR sensors being proposed in this paper provide a much higher value of FOM_localized_ than those reported previously.

## 4. Conclusions

The theoretical investigation of a novel nanostructure geometry, consisting of a periodic array of Au crossed-bowtie nanostructures interspaced with Au nanocrosses, as a LSPR-based refractive index sensor was presented. Several geometric parameters were varied to study their distinct effects on the performance of said nanostructure geometry. Smaller gap distance between the nanopillars gave the highest sensitivity and figure of merit, with high localized electric-field enhancement. For variation in the nanocross pillar width and triangular pillar side length, there was a trade-off between high sensitivity and high figure of merit. While increasing the nanocross pillar width led to a decrease in sensitivity and higher figure of merit, increasing the triangular pillar side length results in a higher sensitivity and lower figure of merit. The Mode 1 and Mode 2 dips of plasmon resonance were primarily observed for the nanostructure geometry. Increasing the triangular pillar side length leads to red-shift of the resonance wavelengths, whereas increasing the nanocross pillar width causes a blue-shift of the resonance wavelengths. Changes in the surrounding refractive index—bulk change or localized, brings about a red-shift in the reflectance spectra of the nanostructure geometry. The response of the nanostructure geometry was studied for a wide spectrum, and thus can be suitably tailored for multiple applications. By varying the geometrical parameters of these plasmonic nanostructures, the plasmon resonance wavelengths of these LSPR sensors can also be tunably varied for a wide spectral range. The geometrical parameters of the plasmonic nanostructures—such as nanocross pillar width, the triangular pillar side length, and edge-to-edge gap distance—were varied to enable these LSPR-based sensors to operate at communication wavelengths (1310 nm and 1550 nm). This nanostructure geometry (‘*W*’ = 100 nm, ‘*L*’ = 90 nm, and ‘*d*’ = 6 nm) has a maximum bulk sensitivity of 1000 nm RIU^−1^ with FOM_bulk_ = 4.76 RIU^−1^, and a localized sensitivity of 70 nm/nm (for Δ*n* = 0.2) with FOM_localized_ = 0.33 nm^−1^. This sensitivity of localized refractive index sensing is the highest reported thus far for LSPR-based sensors. The highest value of bulk sensitivity of 1753 nm RIU^−1^, with a figure of merit for bulk sensing (FOM_bulk_) of 3.65 RIU^−1^, was obtained for certain optimized geometrical parameters of these plasmonic nanostructures (‘*W*’ = 100 nm, ‘*L*’ = 160 nm, and ‘*d*’ = 6 nm). This value of bulk sensitivity is higher in comparison to previously proposed LSPR sensors based on plasmonic nanopillars and nanocrosses. Hence, the plasmonic nanostructures being proposed in this paper can be employed for highly sensitive detection of biomolecules of interest.

## Conflicts of interest

There are no conflicts to declare.

## Supplementary Material
